# Imperfections in transparency and mimicry do not increase predation risk for clearwing butterflies with educated predators

**DOI:** 10.1002/ece3.70307

**Published:** 2024-09-20

**Authors:** Justin Yeager, Abigail Robison, Cordon D. Wade, James B. Barnett

**Affiliations:** ^1^ Grupo de Investigación en Biodiversidad, Medio Ambiente y Salud (BIOMAS), Facultad de Ingenierías y Ciencas Aplicadas Universidad de Las Américas Quito Ecuador; ^2^ Dirección General de Investigación y Vinculación Universidad de Las Américas Quito Ecuador; ^3^ School of Natural Sciences Trinity College Dublin Dublin Ireland

**Keywords:** aposematism, camouflage, clearwing butterfly, generalized avoidance, multiple defenses, transparency

## Abstract

Transparency is an intuitive form of concealment and, in certain butterflies, transparent patches on the wings can contribute to several distinct forms of camouflage. However, perhaps paradoxically, the largely transparent wings of many clearwing butterflies (Ithomiini, Nymphalidae) also feature opaque, and often colorful, elements which may reduce crypsis. In many instances, these elements may facilitate aposematic signaling, but little is known of how transparency and aposematism may interact. Here, we used field predation trials to ask two main questions regarding camouflage and signaling in Ithomiini clearwings. In Experiment 1, we focused on camouflage to ask where being transparent may have an advantage over being opaque. We predicted that, as a single opaque pattern can only match a limited range of backgrounds, transparent wings would offer more effective concealment, and experience lower predation risk, over a wider range of backgrounds colors (i.e., green vs. brown substrates) and behaviors (i.e., perched vs. flying) than opaque wings. In Experiment 2, we focused on the effect conspicuous opaque colors may have on clearwing survival. We predicted that although salient signals may increase detectability, those commonly associated with toxic Ithomiini clearwings would not increase predation risk. Both experiments were conducted among educated predators within the natural range of Ithomiini clearwings and we found predation rates to be very low. In Experiment 1, we found some marginal evidence to suggest that opaque, but not transparent, butterflies may suffer increased predation during flight, whereas in Experiment 2, we found equal survival across all model prey types regardless of coloration. Taken together we suggest that any loss of camouflage due to conspicuous coloration may be compensated by aversive signaling, and that educated predators may broadly generalize across a wide range of known and novel clearwing phenotypes.

## Graphical abstract

Clearwing butterflies seemingly balance camouflage (conferred by transparent wing elements) with alternate anti‐predatory strategies including warning coloration, paradoxically resulting in a diversity of efficient, yet imperfectly transparent phenotypes.

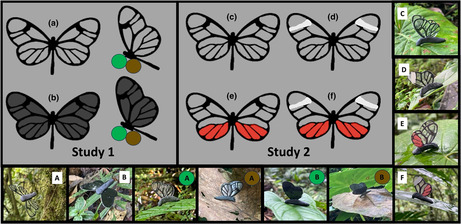

## 1 INTRODUCTION

Camouflage encompasses a diverse set of commonly utilized mechanisms which aid in diminishing the likelihood of detection and subsequently attenuate threats related to predation (Cuthill, 2019). Animal transparency has long been recognized for its potential to provide effective concealment for prey species, where it has largely been considered conceptually synonymous with background matching. Over time this definition has broadened, and it is now increasingly suggested that variation in the degree of transparency, or the combination of opaque and transparent elements, may facilitate several distinct forms of camouflage including background matching (Arias et al., 2020; Michalis et al., 2017), edge and surface disruption (Arias et al., 2021; Costello et al., 2020), and edge diffusion (Barnett et al., 2020; Webster et al., 2015). Yet many questions remain, as we aim to improve our understanding of where transparency may have an advantage over opaque forms of concealment and how transparency may interact with different defensive strategies, such as aposematism.

Transparency is an intuitive way to achieve accurate camouflage via background matching as it is the actual background that can be viewed through the transparent regions. In a series of field predation studies, transparent patches on the wings of model butterflies have been shown to provide effective camouflage, with (mostly) transparent wings surviving as well as models lacking wings entirely and outperforming opaque (cryptic, but homogeneously, colored) wings (Arias et al., 2020; Michalis, 2017). As the opaque treatments in these studies lacked patterning, which is often vital for effective concealment (Cuthill, 2019; Cuthill et al., 2005; Michalis et al., 2017; Stevens & Merilaita, 2011), transparency cannot be said to be superior to opaque background matching per se. However, transparency may be hypothesized to offer two main advantages over opaque coloration. First, transparent camouflage may be effective across a greater variety of background types when compared to any single opaque pattern. Second, transparency may be effective for species active in transparent media (e.g., while flying or swimming) where any opaque camouflage may be broken by changing viewing angles, traversing heterogeneous habitats, motion, or various depth cues.

Recent exploration into transparency as camouflage has largely focused on studies of clearwing or glasswing butterflies found in tropical regions of Central and South America. Many species have highly transparent wings and are diurnal, being at risk of detection during flight and when stationary on substrates, such as leaves and flowers, that can vary greatly in both color and patterning. However, transparency is often imperfect, either being better defined as translucency (Arias et al., 2019), or with patterns that combine both transparent and opaque components (Michalis, 2017). For example, in many Ithomiini (Nymphalidae) species (e.g., *Greta oto* & *G. andromica*, *Ithomia salapia* & *Brevioleria seba*) the transparent wings are outlined by black markings and accompanied by a bright white forewing stripe (Arias et al., 2019; Corral‐Lopez et al., 2021; Michalis, 2017).

Imperfect transparency can still be an effective means of achieving camouflage (Arias et al., 2021; Barnett et al., 2020; Costello et al., 2020; Webster et al., 2015), and the black outline of Ithomiini clearwings appears to have minimal impact on detectability (Michalis, 2017). However, the white stripe does seem to compromise the cryptic effects of transparency (Michalis, 2017) suggesting that it may serve an alternative function, such as aposematism and/or sexual signaling. Indeed, many imperfectly transparent Ithomiini butterflies are chemically defended, likely having evolved transparency from a toxic and conspicuously colored ancestor (McClure et al., 2019). Consequently, aposematism may also play an additionally important role in clearwing butterfly coloration. Accordingly, the presence of white stripes reduces the number of attacks from experienced predators (M) but increases predation rates from naïve predators (Michalis, 2017), yet clearwings are still less detectable than closely related opaque and conspicuously colored species (Arias et al., 2019) suggesting that they may gain an advantage from the combination of camouflage and an aposematic signal (McClure et al., 2019). Taken together these studies, therefore, suggest either a balance or a trade‐off, between camouflage and aposematism (Arias et al., 2019; McClure et al., 2019; Michalis, 2017; Willmott et al., 2017), which is yet to be studied in this system or with the butterflies' natural predators.

Moreover, in highly diverse regions like the Neotropics, predators may be presented with a wide arrange of different prey phenotypes that may complicate important fitness‐influencing foraging decisions. This raises the question of how predators may distinguish between, or whether they generalize across, variable or multicomponent signals within the prey community. For example, in clearwing butterflies, many of which are considered Müllerian mimics (Arias et al., 2019; McClure et al., 2019; Willmott et al., 2017), it is unknown whether predators respond to individual cues or more generally to a broader toxic clearwing motif. Foraging decisions that may be contingent on the composition of the local predator community, as well as their prior experience with different clearwing butterfly phenotypes (McClure et al., 2019; Willmott et al., 2017).

Here we use artificial prey experiments to examine how transparency and opaque coloring affect the survival of butterflies in a highly diverse Andean cloud forest where the local community is comprised of both toxic and non‐toxic clearwing species. Firstly, in Experiment 1, we manipulate the background to assess how the survival of transparent and opaque winged models varies across multiple distinct microhabitats (background substrates), including when at rest on vegetation and when flying with no adjacent substrate. We predict that because a static opaque pattern cannot match multiple or variable backgrounds, the survival of opaque targets will differ significantly between background types, whereas transparency will be equally effective in all microhabitats. Then, in Experiment 2, we manipulate the color of opaque pattern components to examine whether conspicuous signals increase predation risk and if avoidance behavior is generalized to novel combinations of signal components. We predict that conspicuous opaque coloring will reduce camouflage in all cases, but only colors associated with non‐toxic species will increase predation rates compared to camouflage alone (i.e., transparent without opaque coloring).

## 2 METHODS

Experiments were conducted in an area of primary Andean cloud forest outside the town of Mindo (Pichincha Province, Ecuador), a site with a high abundance of birds and clearwing butterflies. The two predation experiments were conducted on the same transect between September and November 2023 (Experiment 1: September 28 to October 15, 2023. Experiment 2: October 28 to November 22, 2023), toward the end of the dry season.

**FIGURE 1:**
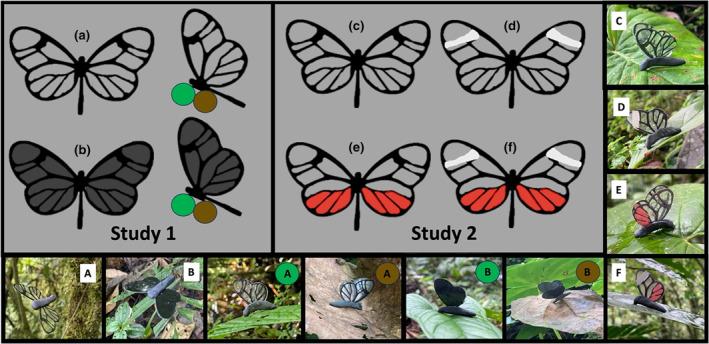
Examples of the treatments used in the predation studies. Left: Experiment 1—transparent (a) and partially opaque (b) with open (flying—left) and closed (perched—right) wings. Perched models were placed on green or brown substrates, whereas flying models were suspended in the air. Right: Experiment 2—transparent (c), ‘aposematic’ transparent (d), conspicuous non‐defended (e), and novel combined phenotype (f). Note that a gray background is used to illustrate the white forewing bands on models, and all light gray regions that match the background color were fully transparent in model butterflies, also the red‐appearing portions on models E/F (gray background) appear pink when printed on transparent sheets (see perimeter photos for in situ examples).

Predation studies followed a well‐established protocol in which artificial butterfly‐like models were placed in blocks along transect through the forest, where they were exposed to attacks from wild predators (Chouteau et al., 2016; Dell'Aglio et al., 2016). Our models approximated an idealized form of an Ithomiini butterfly, with transparent wings adorned with various opaque markings (Figure 1). The wings were made from transparent acetate paper (Superpaco®, A4 size sheets) and for all treatments we printed the opaque outline/venation pattern found in many clearwing species with opaque black ink. Both acetate and butterfly wings have a similar transmission profile, being highly transparent for light between 400 and 700 nm (human visible) but absorbing a small percentage of UV (300–400 nm) light (Binetti et al., 2009; Gomez et al., 2021; Herrnsdorf et al., 2010). The body was made from black modeling clay (Pelikan®, Plastilina) that was molded around a galvanized metal structure that afforded greater rigidity and allowed for secure attachment of the model to the substrates.

In Experiment 1, we were interested in how viewing conditions affected the survival of opaque and transparent targets using three common signaling environments: flying and two common substrates (green and brown). We therefore created two types of butterfly model that differed in degree of wing transparency. A *transparent* form with the unmodified, and uncolored, acetate and an *opaque* form where the transparent regions were printed over with translucent black ink. To make the two treatments, prior to printing, the transparent regions were assigned an alpha (transparency) value of 0.0 (*transparent* being fully transparent) and 0.9 (*opaque* being 10% transparent), this allowed the black outline to visible on both the *transparent* and *opaque* targets. We presented these two forms on three different substrates ~1–2 m above the forest floor: these include perched on green and brown leaves to simulate butterflies at rest, and suspended in the air, using lightweight green fishing line (Faith Carp Tackle) tied to branches, to simulate butterflies in flight. Perched models had their wings folded together, whereas flying models had their wings held apart and flat. This created six treatments, *transparent* and *opaque* butterflies perched on *green* and *brown* leaves and suspended as if in *flight* (Figure 1a,b).

**FIGURE 2:**
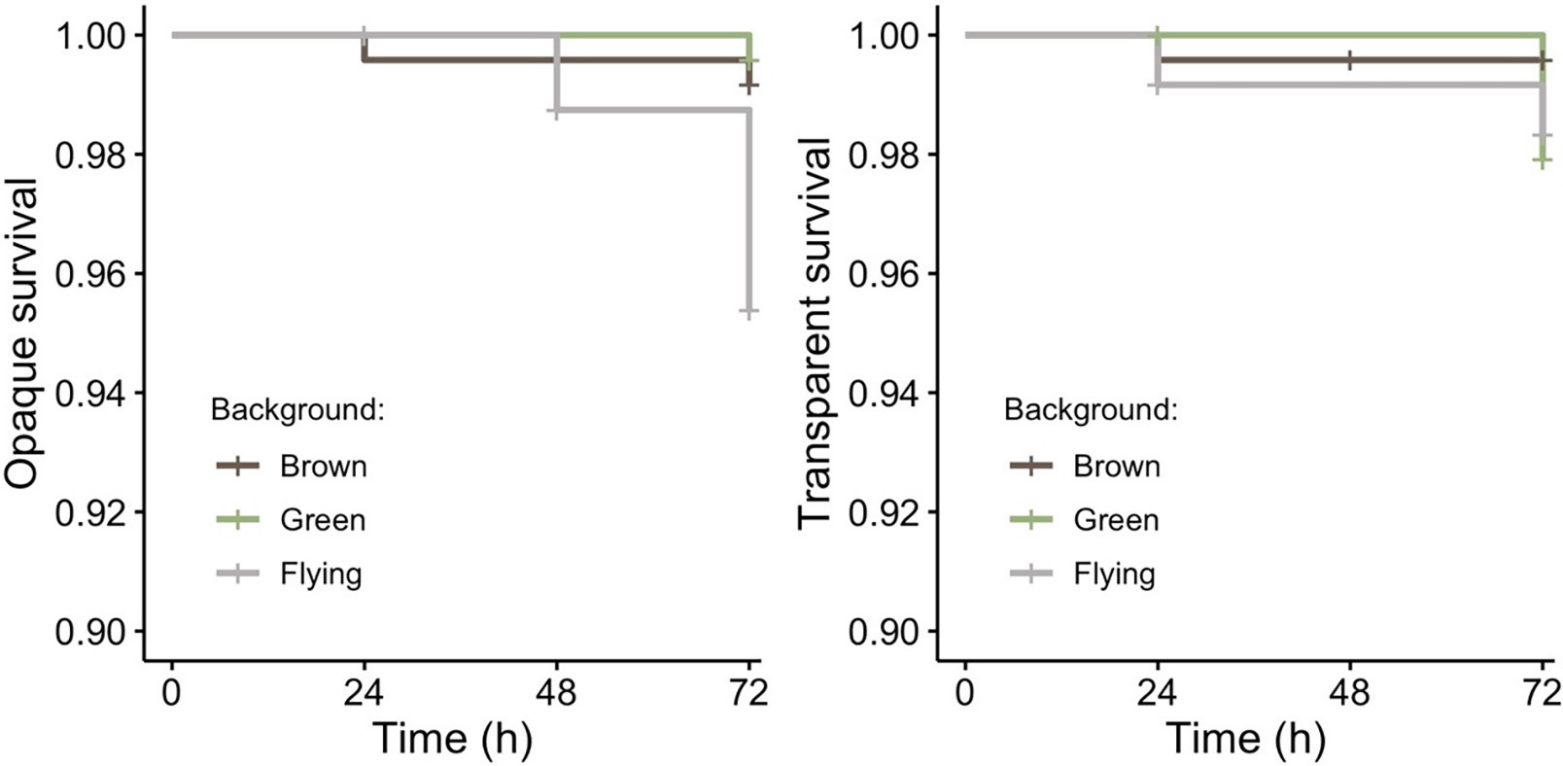
Experiment 1 survival rates for opaque (left) and transparent (right) butterflies across the three backgrounds (brown = perched on brown leaves, green = perched on green leaves, flying = suspended with no adjacent substrate) against bird predators. Left: Survival over 72 h of the opaque butterflies, there was a marginal effect of background with the highest rate of predation on flying butterflies. Right: Survival over 72 h of the transparent butterflies, there was no effect of substrate on predation risk. All treatments had an opaque black outline (see Figure 1a,b).

In Experiment 2, we were interested in how highly contrasting opaque colors affect predation risk to ask whether predators may generalize avoidance behavior across known and novel signals. We created four model types: (1) transparent (Figure 1c), (2) transparent with a white band on the forewing (Figure 1d) characteristic of several aposematic Ithomiini butterflies commonly found at our study site (e.g., *Greta andromica*), (3) transparent with conspicuous pink patches on the hindwing (Figure 1e), a pattern consistent with sympatric non‐toxic clearwings (e.g., *Cithaerias pireta*) but not found associated with toxic Ithomiini clearwings at our study site, and (4) a novel morph which had both the characteristic aposematic white band on the forewing as well as the conspicuous (but not defended) pink patch on the hindwing (Figure 1f). This final form therefore featured a strongly conspicuous, yet highly imprecise ‘mimetic’ phenotype which allows us to examine the interacting effects of the two conspicuous colors. All models in Experiment 2 were attached perched, with their wings closed, on green leaves ~1–2 m above the forest floor.

For both experiments, we used a randomized block design with 40 independent blocks, each 20 m long. Blocks were set along a single ~3.8 km transect and were spaced to be a minimum of 20 m apart. The topography of the trail ensured that consecutive blocks were not visible from one another. Within each block, models were placed ~1 m off the trail with the side of the trail (left or right), the order of treatments, and distance from the start of the block all randomized.

In Experiment 1, each block contained two models from each of the six treatments and in Experiment 2, each block contained three models from each of the four treatments, resulting in a total of 12 butterflies per block and 480 models per transect in both experiments. Each experiment was replicated, three (Experiment 1) or four times (Experiment 2), with a minimum of 3 days between trials. Therefore, a total of 1440 models (240 per treatment) were used in Experiment 1 and a total of 1920 models (480 per treatment) were used in Experiment 2.

Models were visually inspected for attacks daily for 72 h. Models were scored as attacked by wild predators by the presence of dentition, beak, or other visible indentations into the clay, the total absence of clay, or the removal of wings from model bodies when not attributable to rain or falling debris. Attacks were confirmed by consensus between a minimum of two independent reviewers (J.Y., A.R., and C.D.W.) that the indentation/mark was caused intentionally by a vertebrate predator and unlikely the result of an invertebrate or alternative scenario such as falling debris. Attacked models were left in‐situ until the end of the experiment, but as all models were placed to be independent of each another their presence should not have affected our data.

We then analyzed mortality risk for each experiment with a mixed‐effects Cox model, function *coxme*, and checked model assumptions, function *cox.zph*, from R package *coxme* (Therneau, 2015) in R v.4.3.2 (R Core Team, 2023). In Experiment 1, we included the interaction between wing type (*transparent* or *opaque*) and substrate (*green*, *brown*, or in *flight*) and for all analyses, we included the block number and the experimental replicate as random factors. Where relevant we performed pairwise Tukey tests using R package *multcomp* (Hothorn et al., 2008). The analyses were repeated twice, firstly including just the confirmed bird predation events and secondly including all potential predation events (including non‐bird predators, i.e., lizards and rodents, and missing targets). We include the two analyses to allow for a conservative assessment of the confirmed bird‐only data, while also acknowledging that our exclusion criteria may have removed some legitimate predation events, in which case non‐visual predation or mechanical loss of targets should not bias the underlying effect of color.

## 3 RESULTS

In Experiment 1, 50 of the 1440 targets (3.5%) showed evidence of attempted predation, of which 24 (24/50 events) displayed features suggestive of bird attacks. When analyzing only the confirmed bird predation events we found a marginally significant interaction between wing type and background (*χ* ^2^ = 6.30, df = 2, *p* = .043). As any differences in survival between opaque and transparent models was contingent on background we analyzed the effect of background on the survival of opaque and transparent butterflies separately (Figure 2). There was no significant effect of background for the transparent butterflies (*χ* ^2^ = 3.26, df = 2, *p* = .196), but background type did affect the survival of opaque butterflies (*χ* ^2^ = 12.78, df = 2, *p* = .002). Pairwise Tukey comparisons for the opaque butterflies showed no significant difference between the green and brown backgrounds (*z* = −0.57, *p* = .832), whereas there was a non‐significant, but marginal trend for predation on opaque models to be higher when flying than when perched on either the brown (z = 2.25, *p* = .059) or green (z = 2.33, *p* = .050) backgrounds.

Conversely, when analyzing all of the predation events (including non‐avian attacks and missing targets), the interaction between wing type and background was non‐significant (*χ* ^2^ = 4.63, df = 2, *p* = .099) and the main effect of wing type was non‐significant (*z* = 0.02, *p* = .98). There was also no effect of background on the survival of either the opaque (*χ* ^2^ = 1.08, df = 2, *p* = .584) or transparent (*χ* ^2^ = 4.01, df = 2, *p* = .135) butterflies.

**FIGURE 3:**
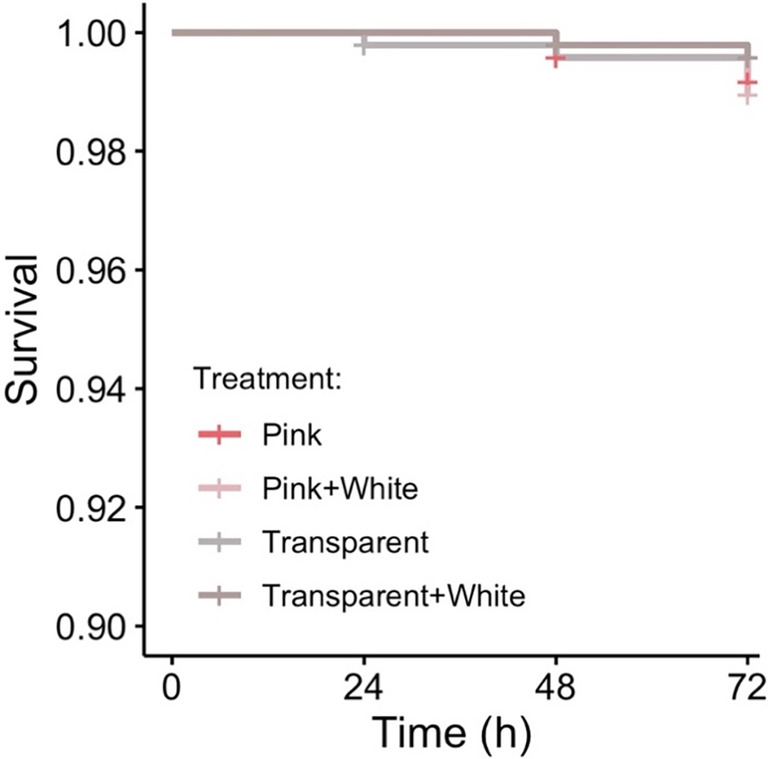
Experiment 2 survival rates for transparent butterflies against bird predators (Pink = transparent with pink hindwings; Pink + White = transparent with a white stripe on the forewing and pink hindwings; Transparent (fully transparent); Transparent + White = transparent with a white stripe on the forewing). There was no effect of coloration on predation risk. All treatments had an opaque black outline (see Figure 1c–f).

In Experiment 2, predation rates were also low with 16 of the 1920 models (0.8%) exhibiting evidence of attempted predation, the majority of which (13/16 events) could be confidently identified as originating from birds. There was no significant effect of treatment on the rate of survival both when including all predation events (*χ* ^2^ = 1.42, df = 3, *p* = .701) and when restricting analysis to confirmed bird attacks (*χ* ^2^ = 2.10, df = 3, *p* = .552; Figure 3).

## 4 DISCUSSION

We assessed phenotype‐specific predation risk across model butterflies which exhibited variations on a clearwing butterfly motif. In Experiment 1, we were interested in how variable viewing conditions affected the survival of transparent and opaque butterflies (i.e., survival on different substrates and when flying). In line with our predictions, we found there to be a slight trend for the opaque, but not the transparent, butterflies to be attacked more frequently when in an open‐winged posture and suspended above the substrate, than when perched with closed wings on either green or brown leaves. This effect was only evident when focusing on confirmed bird attacks, and pairwise comparisons were marginal. Yet this trend for differences in substrate specific risk warrants further study to examine whether transparency can be more effective than opacity at providing camouflage during flight.

It is also important to note, however, that overall attack rates were low, and although we do not directly compare between transparent to opaque butterflies, this does contrast with studies on Ithomiini clearwing butterflies conducted outside of their natural range with naïve avian predators. In these studies, transparent butterflies do have significantly lower detectability and greater survival than opaque butterflies (closed wings, Arias et al., 2019; Michalis et al., 2017).

In Experiment 2, we examined the effect of conspicuous opaque colors on the survival of clearwing butterflies. Here, contrary to our predictions, there was no evidence that the addition of opaque colors to transparent wings affected predation risk. This similarly contrasts with previous research where the addition of a white stripe to the forewings increased predation risk from naïve birds in Europe (Michalis, 2017), but reduced predation risk from educated predators in Colombia (Corral‐Lopez et al., 2021). Unlike previous work, however, we also manipulated the presence of pink markings on the hindwings in a manner reminiscent of a local non‐toxic species (*C. pireta*—transparent with pink on the hindwings) and to create a novel, seemingly conspicuous, phenotype not represented in any local species (transparent with a white stripe on the forewing and pink on the hindwings). Yet, despite including ostensibly poorly camouflaged color patterns, we find close to 100% survival for all combinations of white and pink markings regardless of whether it mimicked local defended, local palatable, or novel (and imperfectly mimetic) clearwing phenotypes.

Low predation could be explained if there are simply few predators at our study site, or by our models being placed where they were not often encountered. Alternatively, birds may not have recognized the clay as a potential food source, or our inclusion criteria for attacks may have been too conservative in removing ambiguous marks that could not be confidently assigned to a predator. However, although we cannot completely dismiss these possibilities, our experiments followed a very well‐established protocol, and so we believe their impact to be minimal. Firstly, our study was conducted in the primary forest around Mindo, Ecuador, a world‐renowned birding destination, with an abundance of lower story species that are frequently observed foraging along the trails and from feeding platforms (JY, AR and CW *pers. obvs*.). Moreover, studies using the same experimental paradigm, but with differently colored targets, have found significant results with both conspicuous and cryptic butterflies (e.g., *Heliconius* spp.) in similar habitats. This includes the use of clay bodies, the 72 h deployment time, perched and suspended models, and a sample size of ~200–400 per treatment (e.g., Dell'Aglio et al., 2016; Finkbeiner et al., 2014), and suggests that low predation rates result from local ecological factors rather than methodological constraints.

Taken at face value, our low predation rates suggest that all our phenotypes were highly, and equally, successful at mitigating predation risk at our study site. As, with most artificial prey experiments, we are unable to differentiate survival due to a lack of detection from that arising from detection and subsequent avoidance, it is plausible that all models were highly cryptic and remained undetected. However, given that white markings are aposematic (Corral‐Lopez et al., 2021) and reduce camouflage for naïve predators (Michalis, 2017), detection and avoidance for at least some of our experimental treatments seems likely. Ithomiini clearwings are chemically defended (McClure et al., 2019), and while our predation rates are low (3.5% and 0.8%) they are still comparable to those recorded for conspicuously colored aposematic *Heliconius* spp. (<5%) in equivalent field‐based predation experiments with educated predators (Chouteau et al., 2016; Finkbeiner et al., 2014; Merrill et al., 2012; Ogilvie et al., 2021). As such, we suggest that predation rates for clearwing butterflies may truly be very low under the natural conditions found at our study site.

Indeed, as camouflage and aposematism are not mutually exclusive (Arias et al., 2019; Kikuchi et al., 2023), it may be that even if imperfect transparency does reduce camouflage (Arias et al., 2019; Michalis, 2017) an increase in detectability can be offset by the benefits conferred by aposematism (McClure et al., 2019). Transparency and warning signals are both predicted to be effective anti‐predator strategies, and camouflage and conspicuous signals can provide equal survival under field conditions (Carroll & Sherratt, 2013; Ogilvie et al., 2021; Seymoure et al., 2018). As predation risk did not vary between our treatments, we find no evidence of a trade‐off between strategies, but our data fall short of being able to conclude there is a balance between crypsis and additional phenotypic‐specific benefits. We do, however, urge caution when interpreting our results broadly, and use this opportunity to call for further research, especially within the natural range of these butterflies and across different geographic sites where local populations of predators may differ.

Therefore multiple effective sympatric defensive strategies may explain the low attack rates, which may be further explained by a high diversity of clearwing butterfly phenotypes, and suites of predators (Willmott et al., 2017) familiar with them at our study site. In Mindo, Ithomiini clearwings are notably common with a high diversity ~18 species reported (identified as ‘research grade’ through the citizen science platform iNaturalist www.inaturalist.org, 03 April 2024). Many species are Müllerian mimics that vary in the degree of transparency and in the presence of white markings, but mostly share the black outline (e.g., *Greta andromica*, *Ithomia terra*, & *Oleria padilla*) (Beccaloni, 1997; Chazot et al., 2016; McClure et al., 2019). Moreover, red and white are also common aposematic signals which are found on other defended butterflies at our field site (e.g., *Heliconius clysonymus* which is predominantly black with a white forewing stripe and red hindwings; Blow et al., 2023 preprint). Consequently, avian predators may generalize across many different signal components to be cautious of clearwing‐like butterfly motifs generally as well as hesitant to interact with both familiar and novel combinations of signals. Further work is needed to examine whether predators may recognize and avoid salient pattern components, such as opaque coloring, or more cryptic but recognizable elements, for example the black outline, body shape, or transparency itself. Nonetheless, in a region with a high density of transparent clearwing butterfly species, we find no evidence that models with imperfect transparency, or imperfect mimicry suffered higher predation rates by native predators than more highly transparent or aposematic models.

Taken together, our data suggest that in their natural habitat, Ithomiini clearwing butterflies may have a low risk of attack from predatory birds. In Experiment 1, we find some evidence to suggest that transparency may be an effective form of concealment during flight. However, attack rates were too low to draw strong conclusions and further work is needed to address this possibility. In Experiment 2, we found no evidence that the addition of conspicuous opaque colors reduced the survival of our targets despite previous work suggesting such manipulations would increase detectability (Arias et al., 2019; Michalis, 2017). We suggest that this may result from an interaction between camouflage and aposematism (McClure et al., 2019), and that predators may generalize their avoidance behavior to novel signals. However many questions remain in our understanding of signaling and predation risk in this system.

The role of aposematism, and our failure to replicate results from naïve predators, highlights the importance of conducting behavioral studies within the natural ecological, behavioral, and environmental context. As such, caution should be exercised when interpreting ex situ experiments, but studies of naïve predators and humans continue to be an important tool for teasing apart the role of camouflage while removing the effect of aposematism and neophobia. In summary, we suggest that clearwing butterflies utilize a balance between transparency and alternate anti‐predatory strategies (e.g., aposematism or mimicry), and that generalized avoidance is likely across diverse clearwing butterfly phenotypes. However, more research is needed to understand how predators perceive, recognize, and evaluate clearwing butterflies as prey.

## AUTHOR CONTRIBUTIONS

**Justin Yeager:** Conceptualization (lead); data curation (supporting); funding acquisition (lead); investigation (equal); methodology (equal); project administration (equal); resources (equal); supervision (equal); writing – original draft (lead); writing – review and editing (equal). **Abigail Robison:** Data curation (lead); investigation (equal); methodology (supporting); writing – review and editing (supporting). **Cordon D. Wade:** Data curation (lead); investigation (equal); methodology (equal); writing – review and editing (supporting). **James B. Barnett:** Conceptualization (equal); data curation (equal); formal analysis (equal); methodology (equal); writing – original draft (supporting); writing – review and editing (equal).

## FUNDING INFORMATION

JY was funded by UDLA grant 483.A.XIV.24, AR and CW were funded by UDLA seed grants from the UDLA Vicerectorado.

## CONFLICT OF INTEREST STATEMENT

None.

## Data Availability

Data are available on Dryad: https://doi.org/10.5061/dryad.fn2z34v3j.
